# PLZF play as an indirect facilitator of thymic retention for the innate-like T-cells to aquire innate-like functions

**DOI:** 10.1038/s41419-018-1075-y

**Published:** 2018-10-11

**Authors:** Xin Cao, Xiao-xia Ma, Yu-jia Xue, Yan Zeng, Xian-yu Zhang, Ying Lu, Jiang-long Du, Peng Ma, Qiu-yan Chang, Lin-jie Li, Xue-yan Zhou, Kui-zheng Cai, Damian Kovalovsky, Zhong-ren Ma

**Affiliations:** 1 College of Life Science and Engineering, Northwest Minzu University, Engineering & Technology Research Center for Animal Cell, Gansu, China; 2 Experimental Immunology Branch, National Cancer Institute, National Institutes of Health, Bethesda, MD 20892 USA; 3 State Key Laboratory of Veterinary Etiological Biology, Lanzhou Veterinary Research Institute, Chinese Academy of Agricultural Sciences, Lanzhou, 730046 China; 4 Ministry of Education Key Laboratory of Molecular Microbiology and Technology, Nankai University, Tianjin, 300071 China; 5 Key Laboratory of Bioengineering & Biotechnology of State Ethnic Affairs Commission, Lanzhou, 730030 China

Dear editors,

Innate-like T-cells can be placed in-between the adaptive and innate immune systems. The mechanisms that determine the differentiation of innate-like T-cells is not completely understood^1,2^. Innate-like T cells include invariant Natural Killer T-cells (iNKT), which express a αβTCR, and γδNKT cells, which express a γδTCR corresponding to Vδ6.3 and Vγ1.1. Previous study showed that Zbtb16 (PLZF) was necessary for the acquisition of an innate-like phenotype in iNKT and γδNKT cells^3–5^. Absence of PLZF severely impairs iNKT cell development, leading to a reduction of iNKT cell numbers. iNKT cells that develop in PLZF-deficient mice have a naive phenotype, lost the ability to co-express IL4 and IFN-γ, lost the ability to migrate to the liver and preferentially located to lymph nodes^3,4^. Reciprocally, transgenic PLZF expression was sufficient to confer an effector phenotype to T-cells and similar migratory properties as iNKT cells^6–8^. This phenotypic conversion occurred during development and in the absence of agonist selection, indicating that PLZF expression was sufficient to alter the phenotypic characteristics of T-cells^9^. γδΝΚT-cells expressing Vγ1.1 and Vδ6.3/6.4 share characteristics of iNKT cells, and in PLZF-deficient mice Vγ1.1 Vδ6.3 γδT-cells were still present in reduced numbers^9^. Furthermore, our previous study demonstrated that PLZF controled the development of fetal-derived IL-17^+^ Vγ6^+^ γδT-cells^10^. However, how PLZF expression is regulated and how it exerts these functions is not clearly understood. We, therefore, decided to focus our efforts in understanding how PLZF expression provides an innate-like phenotype to iNKT cells and γδNKT cells.

At first we decided to evaluate the regulation of PLZF expression during T-cell development by PLZF-GFP reporter mice. Briefly, the reporter mice were generated by control the expression of transgene eGFP with PLZF regulatory elements in a modified bacterial artificial chromosome^11^. We observed GFP fluorescence in iNKT cells (Fig. S1A). PLZF expression was low in early thymic progenitors (ETP), was slightly upregulated at the DN2a stage of development and turned off after T-cell specification at the DN3, DN4, and DP positive stages (Fig. S1B). We observed that contrarily to adult mice, PLZF expression was abundant in fetal thymocytes at every developmental stage (Fig. S1C). In light of the abundant expression of PLZF that we observed in the fetal thymus, we wanted to evaluate if fetal HSC may be biased to differentiate towards innate-like iNKT and γδNKT cells as compared to adult HSCs. We tested this by performing mixtures of congenic Fetal liver and Adult Bone Marrow chimeras and analyzed if iNKT and γδNKT cells were preferentially derived from fetal or adult precursors. We observed that there was no preferential bias of fetal HSCs to give rise to innate-like cells under these conditions (Fig. S1D–F).

We next tested the possibility of innate-like cells being derived from fetal precursors by performing transplants of neonatal day 1 thymus under the kidney capsule of congenic hosts. In this system, cells from the donor transplanted thymus are progressively replaced by differentiating thymocytes derived from host HSCs. Therefore, analysis of the different T-cell subtypes in the transplant derived from donor cells is indicative of the combined ability of these cells to differentiate and to remain in the thymus. We analyzed how iNKT and γδNKT would differentiate in this system. At first, we confirmed that Vδ6.3^+^ γδTCR (γδNKT) in PLZF-GFP reporter mice presented high CD44 levels and a proportion of them were GFP positive, indicative of PLZF expression (Fig. 1a). We then set-up thymic transplants of day 1 neonatal C57BL.6 thymus (CD45.2) under the kidney capsule of congenic Ly5.2 (CD45.1) mice, and analyzed the presence of Vδ6.3^+^ γδNKT and Vδ6.3^-^ γδT-cells in the transplants that were derived from either donor or host cells. Three weeks after transplantation, approximately 6% of the cells in the transplanted thymus was of donor origin. Donor cells showed an increased proportion of Vδ6.3^+^ γδNKT cells among the γδT-cell population (Fig. 1b). This increase of donor Vδ6.3^+^ γδNKT cells was observed at 3 and 4 weeks after transplantation (Fig. 1c). To our surprise, high CD44 expression was not exclusive to Vδ6.3^+^ γδNKT cells and both donor Vδ6.3^+^ and Vδ6.3^-^ γδT-cells in the transplant, but not those derived from the host, had homogenously high levels of CD44 (Fig. 1d). In correlation with high CD44 levels, donor Vδ6.3^+^ γδNKT cells as well as Vδ6.3^-^ γδT-cells expressed PLZF (Fig. 1e). Similarily to γδNKT cells, donor iNKT cells were preferentially retained in the transplants and had a mature CD44^+^NK1.1^+^ phenotype (Fig. 1f).

**Fig. 1:**
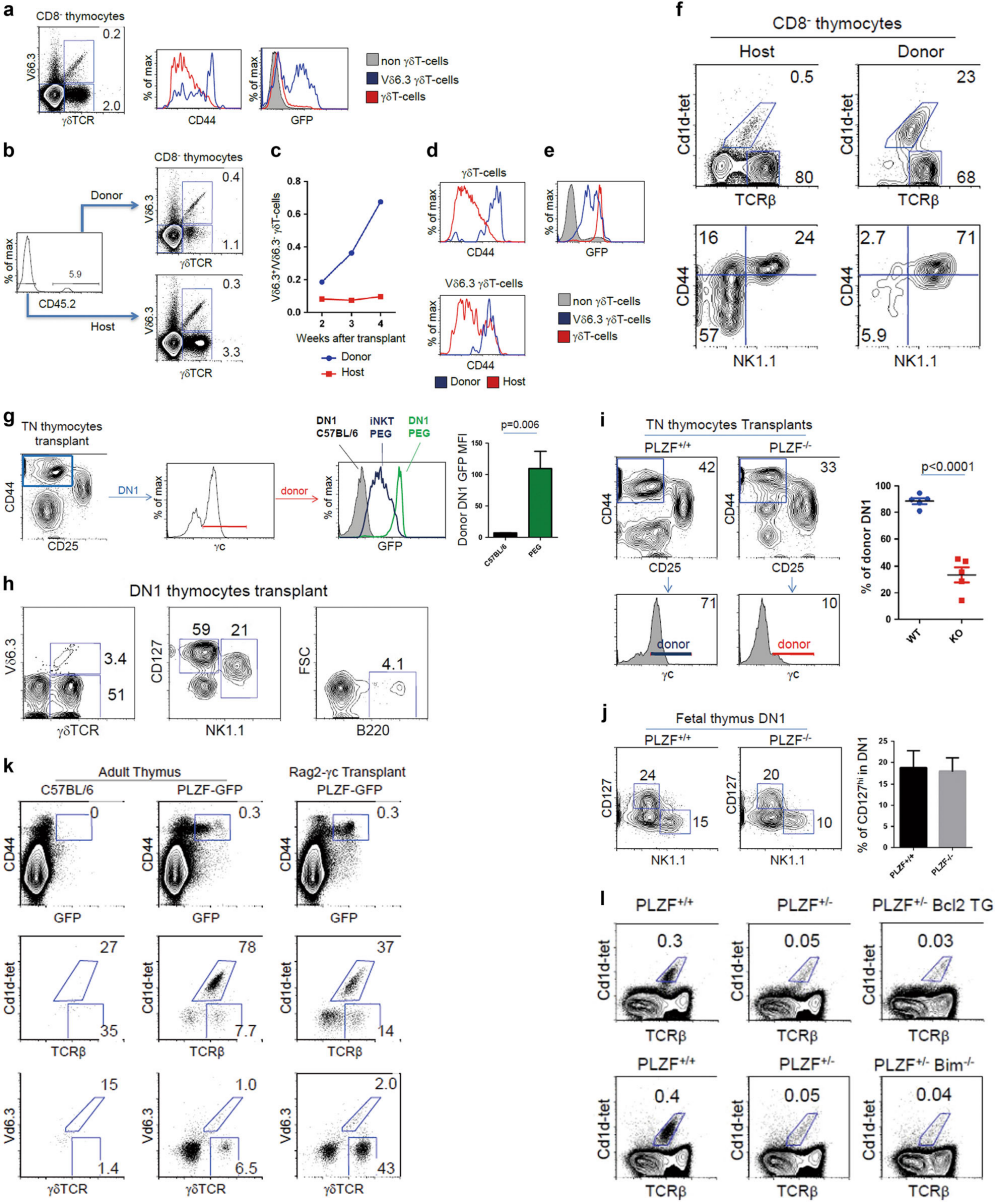
PLZF function in thymic retention as a mechanism for the acquisition of innate-like functions. **a** Proportion of Vδ6.3^+^ and Vδ6.3^-^ γδT-cells in adult PLZF-GFP thymus. CD44 and GFP levels in Vδ6.3^+^ and Vδ6.3^-^ γδT-cells in adult mice. Background staining with the Vδ6.3 antibody is observed in non-γδT-cells. **b** Three weeks after neonatal thymic transplants into congenic hosts. The proportion of Vδ6.3^+^ and Vδ6.3^-^ γδT-cells is shown from host (CD45.2^-^) and donor (CD45.2^+^) cells. **c** Ratio of Vδ6.3^+^/Vδ6.3^-^ cells derived from donor or host cells at different weeks after transplantation. **d** Analysis of CD44 levels in Vδ6.3^+^and Vδ6.3^-^ γδT-cells derived from donor or host cells. **e** Analysis of GFP levels in donor Vδ6.3^+^, Vδ6.3^-^ thymocytes and non-γδT-cells. **f** Increased retention of donor iNKT cells after thymic transplantation into congenic hosts. **g** Triple negative (CD4^−^CD8^−^TCRβ^-^) profile of transplanted thymus into Rag2-γc-deficient hosts. Gate on the DN1 (CD44^+^CD25^−^) population identify cells derived from the donor thymus (γc^+^) and host HSC (γc^-^). Comparison of GFP levels between PLZF-GFP (PEG) and C57BL/6 transplanted thymus gating on the TN, CD44^+^ γc^+^ donor population in relation to the GFP levels of iNKT cells in PLZF-GFP (PEG) mice. **h** Characterization of the PLZF^+^ DN1 population found in the Rag2-γc transplants according to different markers. **i** Analysis of the TN (CD4^−^CD8^−^TCRβ^-^) profile in thymic transplant of day 1 neonates from wild-type or PLZF-deficient thymus into Rag2-γc-deficient hosts. The levels of γc expression in the CD44^+^ population indicates if these cells are donor or host derived. **j** Proportion of CD127^+^ cells among DN1 thymocytes in day 15 fetal thymus from wild-type and PLZF-deficient mice. **k** FACS analysis of adult C57BL/6; adult PLZF-GFP thymus; and PLZF-GFP neonatal transplants into Rag2-γc hosts. Analysis of the proportion of iNKT (Cd1d-tet^+^) and αβT-cells, and of γδNKT (Vδ6^+^) and γδT-cells on CD44^+^GFP^+^ gated cells. **l** Protection from apoptosis in Bim-deficient or Bcl2 transgenic mice does not revert the thymic iNKT phenotype of PLZF heterozygous mice

We were curious to interrogate if the PLZF expressing cells that we observed in the fetal thymus may remain in the adult and maintain PLZF expression if placed under non-competitive conditions in the absence of adult progenitors. To test this, we performed similar thymic transplant experiments of neonatal PLZF-GFP reporter thymus into Rag2/γc double-deficient recipients mice. Analysis of transplants from PLZF-GFP reporter mice showed a population of DN1 thymocytes that expressed high levels of PLZF (Fig. 1g). These CD44^+^PLZF^+^ cells showed a mature phenotype, were heterogeneous, and corresponded mostly to CD127^+^ (IL7Rα^+^), γδT-cells and NK1.1^+^ cells (Fig. 1h).

To evaluate if PLZF confers the ability to thymocytes to remain in the thymus, we set-up transplants of wild-type and PLZF-deficient neonatal thymus under the kidney capsule of Rag2-γc-deficient host mice. One month after transplantation, we observed that PLZF-deficient transplants had a severe reduction of donor CD44^+^ cells, as most of the cells found with this phenotype were derived from the Rag2/γc hosts and were negative for the common gamma chain of the IL-2R (γc) (Fig. 1i). As fetal wild-type and PLZF-deficient thymus had a similar thymic profile, γδT-cells^10^ and proportion of DN1 CD127^+^ thymocytes (Fig. 1j), this led us to postulate that these CD127^hi^ cells, although present in the fetal thymus, were unable to remain in the PLZF-deficient transplants. Independent to these results, we have observed in the PLZF-GFP thymic transplants into Rag2-γc-deficient hosts that among the CD44^+^GFP^+^ populations were many αβT-cells that were not iNKT cells (Cd1d-tetramer^−^) and γδT-cells that were not γδNKT cells (Vd6.3^-^) (Fig. 1k). Using this gating strategy, we were also able to detect these cells in adult PLZF-GFP thymus, although in a lower proportion (Fig. 1k).

As CD44^+^ donor thymocytes from wild-type mice in the thymic transplants did not preferentially incorporate BrDU (data not shown), these results indicated that these DN1 CD127^+^ cells were not actively dividing. Another possible mechanism that could explain the absence of these cells in PLZF-deficient transplants could be by increased apopotosis of cells in the absence of PLZF. However, we think this unlikely due to the inability of bcl2 transgenic expression or Bim deficiency, both which protect from apoptosis, to revert the deficient iNKT phenotype in PLZF heterozygous mice (Fig. 1l).

Altogether, our results suggest that PLZF play a function in the thymic retention of lymphocytes with an innate-like phenotype. As iNKT cells that express the highest levels of PLZF have not yet acquired innate-like features, our results open the possibility of PLZF in mediating thymic retention as a determinant for innate-like differentiation.

## Electronic supplementary material

Figure S1

supplementary figure legends
