# Supplementary figures and images for: PLZF play as an indirect facilitator of thymic retention for the innate-like T-cells to aquire innate-like functions
Source: Cell Death Dis. 2018 Oct 11;9(10):1044. doi: 10.1038/s41419-018-1075-y (PMC6181981; doi:10.1038/s41419-018-1075-y)

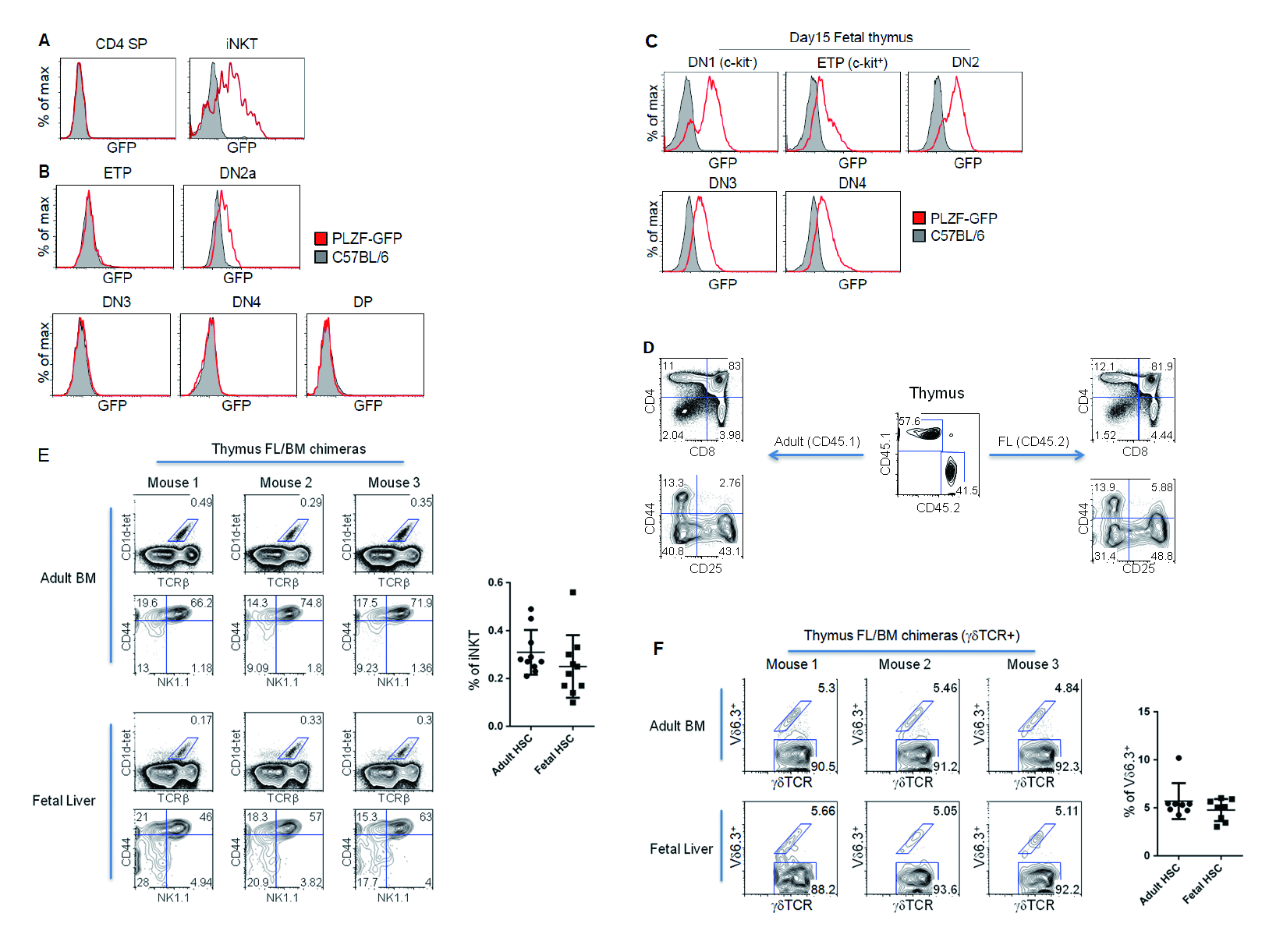

Supplement: Supplementary file 1 — Figure S1 [file 41419_2018_1075_MOESM1_ESM.tif]
