# Supplementary material for: PLZF play as an indirect facilitator of thymic retention for the innate-like T-cells to aquire innate-like functions
Source: Cell Death Dis. 2018 Oct 11;9(10):1044. doi: 10.1038/s41419-018-1075-y (PMC6181981; doi:10.1038/s41419-018-1075-y)
Supplement: Supplementary file 2 — supplementary figure legends [file 41419_2018_1075_MOESM2_ESM.docx]

**Figure S1.** Related to Figure 1.

**(A-B)** GFP levels in PLZF-GFP adult thymus. **(C)**GFP levels in PLZF-GFP fetal thymus.**(D)** Thymic profile of mixed fetal liver (CD45.2^+^Sca1^+^)/bone marrow (CD45.1^+^Sca1^+^) chimeras (2 months). **(E)**Percentage of iNKT from fetal (CD45.2^+^) or bone marrow (CD45.1^+^) origin in mixed fetal liver /bone marrow chimeras (2 months). **(F)** Percentage of γδNKT from fetal (CD45.2^+^) or bone marrow (CD45.1^+^) origin in mixed fetal liver /bone marrow chimeras (2 months).
